# Converging pathways: the interaction between estimated glucose disposal rate and atherogenic index of plasma in MASLD risk stratification

**DOI:** 10.1007/s12072-026-11098-0

**Published:** 2026-05-25

**Authors:** Mingxing Chang, Peipu Shen, Guifang Shen

**Affiliations:** Department of Health Management Center, The Affiliated Hospital of Xuzhou Medical University, 99 West Huai-Hai Road, Xuzhou, Jiangsu China

**Keywords:** Metabolic dysfunction-associated steatotic liver disease, Estimated glucose disposal rate, Atherogenic index of plasma, Insulin resistance, Interaction

## Abstract

**Background:**

Metabolic dysfunction-associated steatotic liver disease (MASLD) is a global health burden linked to insulin resistance and atherogenic dyslipidemia, estimated by the glucose disposal rate (eGDR) and atherogenic index of plasma (AIP), respectively. However, the nature of their combined association—whether additive or interactive—with MASLD risk, and its utility for risk stratification, remains unexplored.

**Methods:**

In this large-scale cross-sectional analysis of 30,143 adults, we calculated eGDR and AIP from routine clinical measures. Their independent and joint associations with ultrasonography-defined MASLD were assessed using multivariable logistic regression, restricted cubic splines (RCS), and interaction analysis. Based on RCS-derived thresholds, participants were categorized into four metabolic phenotypes for combined risk assessment.

**Results:**

Both lower eGDR and higher AIP were independently and nonlinearly associated with greater MASLD risk (p for trend < 0.001). A significant interaction was observed (p < 0.001), revealing a convergent pattern: the protective association of high eGDR appeared attenuated as AIP increased, and the risk disparity attributable to AIP narrowed markedly at low eGDR levels. Participants with the combined low-eGDR/high-AIP phenotype had the highest MASLD odds (OR = 3.42, 95% CI 3.07–3.81). These findings were robust to alternative adjustment strategies and phenotype definitions in sensitivity analyses.

**Conclusions:**

eGDR and AIP exhibit a significant interactive association with MASLD risk, characterized by a convergent pattern: the incremental risk associated with atherogenic dyslipidemia (high AIP) appeared less pronounced under conditions of severe insulin resistance (low eGDR). Integrating these two metrics identifies a distinct high-risk phenotype and may inform future risk stratification strategies. Longitudinal studies are needed to establish causality and clinical utility.

**Supplementary Information:**

The online version contains supplementary material available at https://doi.org/10.1007/s12072-026-11098-0.

## Introduction

Metabolic dysfunction-associated steatotic liver disease (MASLD) has emerged as a leading cause of chronic liver disease worldwide, closely linked to obesity and type 2 diabetes [1, 2]. Its pathogenesis involves systemic metabolic disturbances, notably insulin resistance (IR) and atherogenic dyslipidemia [3]. These pathways interact to promote hepatic steatosis, inflammation, fibrosis, and elevated cardiovascular risk [4, 5].

Composite indices derived from routine clinical parameters offer practical tools for integrated risk assessment. The estimated glucose disposal rate (eGDR) is a validated surrogate for whole-body insulin sensitivity, recently linked to MASLD risk [6, 7]. The atherogenic index of plasma (AIP), calculated from standard lipid measures, reflects atherogenic dyslipidemia and is also associated with MASLD prevalence and severity [8, 9].

While eGDR and AIP have each been independently associated with MASLD, their combined effect, potential interaction, and incremental predictive value remain unexamined. This study aimed to quantify the independent and joint associations of eGDR and AIP with MASLD, test for interaction, and evaluate whether their combination improves risk stratification in a large clinical cohort. This approach allows us to assess whether their association with MASLD is merely additive or reflects a more complex interplay.

## Methods

### Study design and participants

This cross-sectional analysis utilized data from the health examination cohort of the Affiliated Hospital of Xuzhou Medical University, collected between January 2021 and March 2025. We initially identified 40,273 adult participants (age ≥ 18 years). Exclusion criteria were applied sequentially: incomplete data for MASLD diagnosis, primarily due to non-selection of optional abdominal ultrasonography (n = 9,291); missing data on questionnaire (n = 288); age ≤ 17 years (n = 25); pregnancy or lactation (n = 6); excessive alcohol intake (n = 124); meeting criteria for MetALD (n = 287); viral hepatitis (n = 65); history of liver surgery, cirrhosis or cancers (n = 44). After applying these criteria, the final analytical sample comprised 30,143 participants. For individuals with multiple visits during the study period, only data from the first eligible examination were included. The participant selection process is detailed in a flowchart (Supplemental Fig. 1). The study protocol received approval from the Institutional Review Board of the Affiliated Hospital of Xuzhou Medical University (XYFY2023-KL086-01), and the requirement for informed consent was waived for this retrospective analysis of anonymized data.

### Covariates

Demographics, anthropometrics, laboratory parameters, and medical history were obtained from electronic health records. Anthropometric measurements included height, weight, waist circumference (WC) and systolic/diastolic blood pressure (SBP/ DBP). Body mass index (BMI) was calculated as weight in kilograms divided by height in meters squared (kg/m^2^). Fasting blood samples were analyzed in the hospital's central laboratory using standardized automated analyzers, including fasting blood glucose (FBG), glycosylated hemoglobin (HbA1c), total cholesterol (TC), triglycerides (TG), high-density lipoprotein cholesterol (HDL-C), low-density lipoprotein cholesterol (LDL-C), alanine aminotransferase (ALT), aspartate aminotransferase (AST), blood urea nitrogen (BUN), creatinine (Cr), uric acid (UA), albumin, and platelet count (PLT). Medical history included self-reported diagnosis of hypertension, type 2 diabetes. Hepatic steatosis was diagnosed via abdominal ultrasonography using standardized ultrasound scanners (Affiniti70, Philips Medical Systems, USA) based on established ultrasonographic criteria [10].

### Key exposures

The estimated glucose disposal rate (eGDR, mg/kg/min) was calculated using a validated formula: 21.158–(0.09 × WC [cm])–(3.407 × hypertension status [present = 1, absent = 0])–(0.551 × HbA1c [%]). The atherogenic index of plasma (AIP) was calculated as log_10_(TG [mmol/L]/HDL-C [mmol/L]).

### Definitions

MASLD Diagnosis: Hepatic steatosis was diagnosed via abdominal ultrasonography performed by trained radiologists using standard criteria. MASLD is defined by the presence of hepatic steatosis alongside at least one cardiometabolic risk factor, after exclusion of other liver disease etiologies and significant alcohol intake, as proposed by the recently published guidelines [11, 12]. The key cardiometabolic criteria, adapted for the Chinese population, are: (1) BMI ≥ 23 kg/m^2^ or WC ≥ 90/80 cm for males/females; (2) blood pressure ≥ 130/85 mmHg or ongoing antihypertensive treatment; (3) TG ≥ 150 mg/dL (1.70 mmol/L) or triglyceride-lowering treatment; (4) HDL-C ≤ 40/50 mg/dL (1.0/1.3 mmol/L) for males/females or lipid-lowering treatment.

MetALD is defined as steatotic liver disease in individuals with metabolic risk factors who consume moderate to high amounts of alcohol. This includes patients with MASLD who have significant alcohol intake (140–350 g/week for women and 210–420 g/week for men [11, 12]. To maintain clarity and alignment with current consensus definitions, the MetALD population was excluded from this analysis. Based on the updated nomenclature, alcohol consumption was categorized as follows: Mild alcohol use: less than 140 g/week for women and less than 210 g/week for men. Moderate alcohol use: 140–350 g/week for women and 210–420 g/week for men. Excessive alcohol use: more than 350 g/week for women and more than 420 g/week for men.

Smoking status was determined based on self-reported lifetime and current smoking habits. Participants who reported having smoked fewer than 100 cigarettes in their lifetime were classified as never smokers. Those who had smoked more than 100 cigarettes were further categorized into three groups: former smokers, occasional smokers, and daily smokers.

Type 2 diabetes was defined as FBG ≥ 7.0 mmol/L, self-reported physician diagnosis, or current use of glucose-lowering medication [13]. Hypertension was defined as SBP ≥ 140 mmHg and/or DBP ≥ 90 mmHg, self-reported diagnosis of hypertension or current use of antihypertensive drugs [14].

### Statistical analysis

#### Overview of analytical strategy

Our analysis comprised three phases: (1) assessing independent associations of eGDR and AIP with MASLD using separately tailored models; (2) examining their interplay (interaction, exploratory mediation analysis, combined phenotypes assessment) using a unified covariate set; and (3) performing sensitivity analyses to test robustness.

Normality of continuous variables was assessed using the Kolmogorov–Smirnov test and visual inspection of histograms. Variables approximating a normal distribution are presented as mean ± standard deviation, whereas those with skewed distributions are presented as median (interquartile range), and categorical variables as frequency (percentage). Group comparisons were made using Student's t-test, Mann–Whitney U test, or chi-square test, as appropriate. Participants with any missing data for variables in a given multivariable model were excluded from that specific analysis (complete-case analysis). As abdominal ultrasonography was offered as an optional add-on examination within the health check-up package, the primary reason for missing data related to MASLD diagnosis was non-selection of this specific test (n = 9291). To evaluate the potential impact of this missingness, we compared baseline characteristics between participants with and without incomplete data. Reassuringly, the distributions of key covariates (age, sex, BMI, and metabolic biomarkers) were largely balanced between the two groups (Standardized Mean Differences < 0.1), suggesting that the missing data was unlikely to introduce major bias regarding the core exposure-outcome associations (Supplemental Table 1).

#### Multivariable regression models for independent associations

To quantify the independent associations of eGDR and AIP with MASLD, separate multivariable logistic regression models were constructed for each index, analyzed in quartiles (Q1-Q4). The highest eGDR quartile (Q4) and the lowest AIP quartile (Q1) served as reference categories. Three sequential modeling strategies were employed: Model 1: Unadjusted. Model 2: Adjusted for age and sex. Model 3 (fully Adjusted for Independent Association): Additionally adjusted for key potential confounders, while deliberately excluding the constituent variables of the index under analysis to prevent over-adjustment. Specifically: For eGDR models, WC, hypertension, HbA1c, and TC were excluded. For AIP models, TG, HDL-C, and BMI were excluded.

#### Definition of the primary adjusted covariate set for interaction and subsequent analyses

To test for effect modification and perform subsequent integrative analyses (e.g., exploratory mediation analysis, phenotype risk) between eGDR and AIP, an integrated Model 3 was defined. We defined this unified model to ensure consistency when examining the relationship between eGDR and AIP. The selection for the adjusted covariate was guided by the need to control for major confounders while avoiding multicollinearity and over-adjustment for the composite indices. Variance inflation factor (VIF) and condition index diagnostics informed the exclusion of total cholesterol due to collinearity with LDL-C. LDL-C was retained in the final model due to its more established direct role in atherogenesis and cardiovascular risk assessment. The constituent variables of eGDR (WC, HbA1c, hypertension) and AIP (TG, HDL-C) were also excluded a priori. The final primary adjusted covariate set included: age, sex, BMI, FBG, LDL-C, hepatic and renal functions, smoking and drinking status. All subsequent multivariable analyses involving both eGDR and AIP (interaction, exploratory mediation analysis, phenotype association) were adjusted for this consistent set of covariates.

#### Examination of nonlinearity and interaction

Potential nonlinear dose–response relationships of eGDR and AIP with MASLD were examined using restricted cubic splines (RCS) with four knots, based on Model 3 for each index.

To statistically test for interaction (effect modification) between eGDR and AIP, a product term of the mean-centered eGDR and AIP was included in a logistic regression model adjusted for the primary covariate set. The nature of this interaction was further probed using the moderation analysis procedure in Hayes's PROCESS macro for SPSS. Conditional effects were visualized by plotting the predicted probability of MASLD across eGDR values at low (25th percentile) and high (75th percentile) AIP levels.

#### Exploratory mediation analysis

Given the cross-sectional design, this analysis is exploratory and aims to describe a plausible statistical explanatory pathway within the data, not to establish causal mediation. To examine the potential statistical role of AIP in the association between eGDR and MASLD, a simple mediation analysis was conducted using Hayes's PROCESS macro for SPSS, using the primary adjusted covariate set for the model. We used the product-of-coefficients method with bias-corrected bootstrapping with 5,000 resamples to estimate the statistical association. The significance of the indirect association was assessed, and the proportion of the total statistical association aligned with this path was calculated as indirect effect/total effect. All exploratory mediation models were adjusted for the primary covariate set. We interpret the results as suggestive of a potential mechanistic link, acknowledging that reverse causation or unmeasured confounding cannot be ruled out.

#### Analysis of metabolic phenotypes

Main analysis in the full cohort: Building upon the nonlinear dose–response relationships identified in the full cohort, we utilized the inflection points from the RCS curves to define clinically relevant thresholds (eGDR = 8.0 mg/kg/min, AIP = –0.15) for risk stratification. Participants were then categorized into four mutually exclusive metabolic phenotypes based on these thresholds: Phenotype I (high-eGDR/low-AIP), Phenotype II (high-eGDR/high-AIP), Phenotype III (low-eGDR/low-AIP), and Phenotype IV (low-eGDR/high-AIP). Associations of these phenotypes with MASLD were analyzed using logistic regression with Phenotype I as the reference, adjusting for the primary covariate set. The results are presented in Table 4.

Internal validation to address double-dipping: To directly address the risk of overfitting and type I error inflation that can arise from deriving and testing thresholds within the same dataset (“double-dipping”) for the four-phenotype classification, we performed an internal validation by randomly splitting the cohort into a training set (70% of participants) and a validation set (30% of participants). The RCS-derived thresholds were re-estimated in the training set (which yielded nearly identical cut-offs: eGDR = 8.0 mg/kg/min, AIP = –0.15). Participants in both sets were then categorized using these training set-derived thresholds, and the associations with MASLD were assessed independently in the validation set. The training and validation set results are provided in Supplemental Table 4. All other analyses (independent associations, interaction, mediation, RCS curves) were performed in the full cohort, as they do not involve threshold derivation or optimization.

### Sensitivity analyses

To assess the robustness of our key analytical decisions, we performed two sets of sensitivity analyses. First, regarding variable selection in the main multivariable models, we evaluated whether the a priori exclusion of the component variables of eGDR and AIP meaningfully influenced the results. We re-ran the fully adjusted models for independent association (Model 3 for eGDR and AIP respectively) after additionally including WC, HbA1c and hypertension (for the eGDR model) or TG and HDL-C (for the AIP model). Second, regarding the classification of metabolic phenotypes, we tested whether the risk stratification based on RCS-derived inflection points was sensitive to the choice of cut-off value. We re-categorized participants using median splits of eGDR and AIP and repeated the phenotype-based logistic regression analysis using the primary adjusted covariate set. The results of these sensitivity analyses are presented in Supplemental Tables 2, 3, Supplemental Figs. 2–4, respectively.

## Results

### Baseline characteristics of participants

The final analytical cohort comprised 30,143 participants, among whom 14,830 (49.2%) were diagnosed with MASLD. As detailed in Table 1, participants with MASLD exhibited a significantly worse cardiometabolic profile compared to their non-MASLD counterparts. They were more likely to be male and had higher median age, BMI, WC, blood pressure, FBG, HbA1c, TG, LDL-C, liver enzymes (ALT, AST, GGT), and UA (all p < 0.001). Concurrently, they had lower levels of HDL-C. Critically, participants with MASLD had a markedly lower median eGDR (8.91 [6.30—10.02] vs. 10.72 [9.38—11.51] mg/kg/min) and a substantially higher median AIP (0.19 [0.02—0.38] vs. −0.11 [−0.28—-0.08]) compared to those without MASLD (both p < 0.001), highlighting the profound underlying disturbances in insulin sensitivity and atherogenic lipemia.

**Table 1:** Characteristics of the MASLD and non-MASLD groups

Characteristics	Overall (n = 30,143)	Non-MASLD (n = 15,313)	MASLD (n = 14,830)	*p* value
General information				
Male^a^	21,105 (70.01)	9113 (59.51)	11,992 (80.86)	< 0.001
Age, year^b^	49 (38, 57)	48 (37, 57)	49 (39,57)	< 0.001
BMI, kg/m^2c^	25.25 ± 3.50	23.28 ± 2.68	27.29 ± 3.05	< 0.001
WC, cm^c^	85.90 ± 10.76	80.01 ± 9.09	91.98 ± 8.79	< 0.001
SBP, mmHg^c^	126.31 ± 17.62	122.18 ± 17.33	130.57 ± 16.89	< 0.001
DBP, mmHg^c^	78.81 ± 12.56	75.47 ± 11.89	82.26 ± 12.30	< 0.001
Glucolipid metabolism				
FBG, mmol/L^b^	5.27 (4.94, 5.73)	5.14 (4.84, 5.50)	5.44 (5.07, 6.00)	< 0.001
HbA1c, %^b^	5.6 (5.3, 5.9)	5.5 (5.3, 5.7)	5.7 (5.4, 6.0)	< 0.001
TC, mmol/L^c^	4.87 ± 0.99	4.75 ± 0.94	5.00 ± 1.02	< 0.001
TG, mmol/L^b^	1.41 (0.97, 2.12)	1.09 (0.80, 1.52)	1.87 (1.33, 2.71)	< 0.001
HDL-C, mmol/L^b^	1.28 (1.10, 1.50)	1.39 (1.19, 1.62)	1.19 (1.03, 1.37)	< 0.001
LDL-C, mmol/L^c^	3.02 ± 0.80	2.89 ± 0.76	3.16 ± 0.81	< 0.001
Hepatic and renal function				
ALT, U/L^b^	21 (15, 32)	17 (13, 24)	27 (19, 40)	< 0.001
AST, U/L^b^	21 (17, 25)	19 (17, 23)	22 (18, 27)	< 0.001
GGT, U/L^b^	26 (18, 42)	20 (15, 29)	35 (24, 55)	< 0.001
BUN, mmol/L^b^	5.02 (4.25, 5.93)	4.93 (4.14, 5.86)	5.11 (4.38, 6.00)	< 0.001
Cr, μmol/L^b^	69 (59, 78)	67 (56, 77)	71 (62, 79)	< 0.001
UA, μmol/L^b^	332 (272, 394)	302 (249, 359)	364 (306, 425.25)	< 0.001
Index				
eGDR, mg/kg/min^b^	9.86 (7.11, 10.91)	10.72 (9.38, 11.51)	8.91 (6.30, 10.02)	< 0.001
AIP^b^	0.04 (-0.17, 0.26)	−0.11 (−0.28, −0.08)	0.19 (0.02, 0.38)	< 0.001
Behavioral risk factors				
Current smoker^a^	4685 (15.54)	1929 (12.60)	2756 (18.58)	< 0.001
Former smoker^a^	8584 (28.48)	4109 (26.83)	4475 (30.18)	< 0.001
Never smoker^a^	16,874 (55.97)	10,139 (66.21)	6735 (45.41)	< 0.001
Mild drinker^a^	19,266 (63.92)	9234 (60.30)	10,032 (67.64)	< 0.001
Never drinker^a^	10,877 (36.08)	5883 (38.41)	4994 (33.67)	< 0.001

*AIP* atherogenic index of plasma, *ALT* alanine aminotransferase, *AST* aspartate transaminase, *BMI* body mass index, *BUN* blood urea nitrogen, *Cr* creatinine, *DBP* diastolic blood pressure, *eGDR* estimated glucose disposal rate, *FBG* fasting blood glucose, *GGT* γ‐glutamyltransferase, *HbA1c* glycosylated haemoglobin, *HDL-C* high-density lipoprotein cholesterol, *LDL-C* low-density lipoprotein cholesterol, *MASLD* metabolic dysfunction-associated steatotic liver disease, *SBP* systolic blood pressure, *TC* total cholesterol, *TG* triglyceride, *UA* uric acid, *WC* waist circumference

^a^Data are presented as n (%)

^b^Data are presented as median (interquartile range, *IQR*)

^c^Data are presented as mean (SD)

### Independent and nonlinear associations of eGDR and AIP with MASLD

Both lower eGDR and higher AIP were strongly and independently associated with an increased risk of MASLD. A striking dose–response relationship was evident when both indices were analyzed by quartiles (Table 2). Compared to the highest eGDR quartile (Q4), the adjusted odds ratios (ORs) for MASLD in Q3, Q2, and Q1 were 2.25 (95% CI 2.02—2.51), 2.68 (95% CI 2.38—3.02), and 2.91 (95% CI 2.56—3.30), respectively (P for trend < 0.0001). The association with AIP was even more pronounced. Relative to the lowest AIP quartile (Q1), the adjusted ORs increased dramatically across Q2 to Q4, reaching 6.03 (95% CI 5.45—6.68) in the highest quartile—-indicating a more than sixfold elevated risk associated with profound atherogenic dyslipidemia (P for trend < 0.0001).

**Table 2:** Dose–response relationship between eGDR and AIP quartiles and the risk of MASLD

	No	Prevalence of MASLD (%)	Model 1 OR (95% CI)	Model 2 OR (95% CI)	Model 3 OR (95% CI)
eGDR quartiles					
Q4	7497	10.54	Ref	Ref	Ref
Q3	7563	46.66	7.43 (6.81–8.10)	8.40 (7.64–9.22)	2.25 (2.02–2.51)
Q2	7517	64.88	15.68 (14.37–17.12)	19.37 (17.57–21.36)	2.68 (2.38–3.02)
Q1	7566	74.46	24.76 (22.63–27.09)	33.87 (30.51–37.59)	2.91 (2.56–3.30)
P for trend	< 0.0001				
AIP quartiles					
Q1	7629	17.05	Ref	Ref	Ref
Q2	7545	39.13	3.13 (2.90–3.37)	2.82 (2.61–3.04)	1.83 (1.67–2.01)
Q3	7641	60.87	7.57 (7.02–8.16)	6.60 (6.11–7.13)	3.10 (2.82–3.41)
Q4	7328	80.87	20.56 (18.92–22.35)	17.59 (16.14–19.17)	6.03 (5.45–6.68)
P for trend	< 0.0001				

Model 1: unadjusted. Model 2: adjusted for age and sex. Model 3: fully adjusted for independent association. For eGDR models, WC, hypertension, HbA1c, and TC were excluded; and for AIP models, TG, HDL-C, and BMI were excluded

*AIP* atherogenic index of plasma, *CI* confidence interval, *eGDR* estimated glucose disposal rate, *MASLD* metabolic dysfunction-associated steatotic liver disease, *OR* odds ratio

**Fig. 1:**
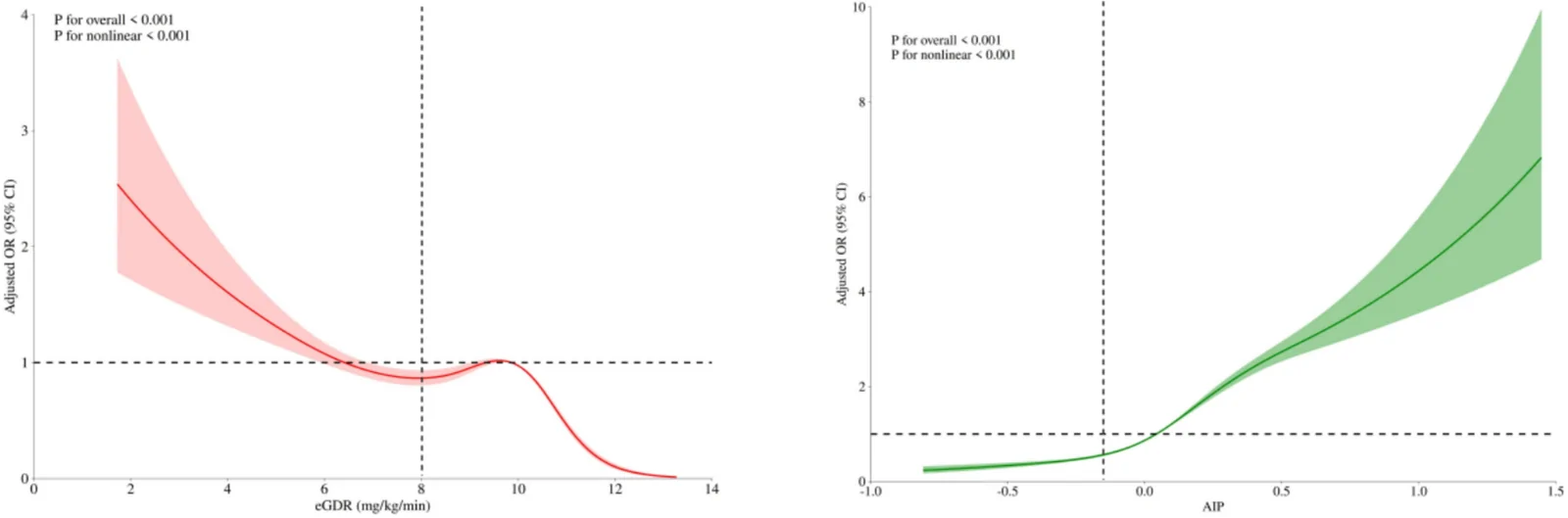
Dose–response curve of eGDR, AIP and MASLD. All curves were adjusted for the integrated Model 3 (age, sex, BMI, FBG, LDL-C, hepatic and renal functions, smoking and drinking status). *AIP* atherogenic index of plasma, *eGDR* estimated glucose disposal rate, *MASLD* metabolic dysfunction-associated steatotic liver disease

When modeled as continuous variables, per IQR decrease in eGDR and each IQR increase in AIP remained significantly associated with MASLD risk after full adjustment (eGDR: OR 1.78, 95% CI 1.23—2.58; AIP: OR 9.50, 95% CI 8.32—10.84) (Table 3). RCS analyses confirmed these were nonlinear associations (both P for nonlinearity < 0.001; Fig. 1). The risk of MASLD increased steeply below an eGDR of approximately 8.0 mg/kg/min and above an AIP of −0.15, identifying potential thresholds for risk stratification. These inflection points remained visually consistent in sensitivity analyses that additionally adjusted for the constituent variables of each index (Supplemental Table 2; Supplemental Fig. 2).

**Table 3:** Logistic regression analysis for the interaction between eGDR and AIP

	Model 1		Model 2		Model 3	
	OR (95% CI)	*p* value	OR (95% CI)	*p* value	OR (95% CI)	*p* value
eGDR (per 1-IQR decrease)	3.90 (3.69–4.10)	< 0.001	4.43 (4.17–4.69)	< 0.001	1.78 (1.23, 2.58)	0.004
AIP (per 1-IQR increase)	4.42 (4.21–4.65)	< 0.001	4.35 (4.13–4.58)	< 0.001	9.50 (8.32, 10.84)	< 0.001
eGDR × AIP interaction	1.20 (1.14–1.27)	< 0.001	1.24 (1.18–1.31)	< 0.001	1.17 (1.10, 1.24)	< 0.001

Model 1: unadjusted. Model 2: adjusted for age and sex. Model 3 (the integrated model 3): adjusted for age, sex, BMI, FBG, LDL-C, hepatic and renal functions, smoking and drinking status

*AIP* atherogenic index of plasma, *CI* confidence interval, *eGDR* estimated glucose disposal rate, *MASLD* metabolic dysfunction-associated steatotic liver disease, *OR* odds ratio

### Interaction between eGDR and AIP

**Fig. 2:**
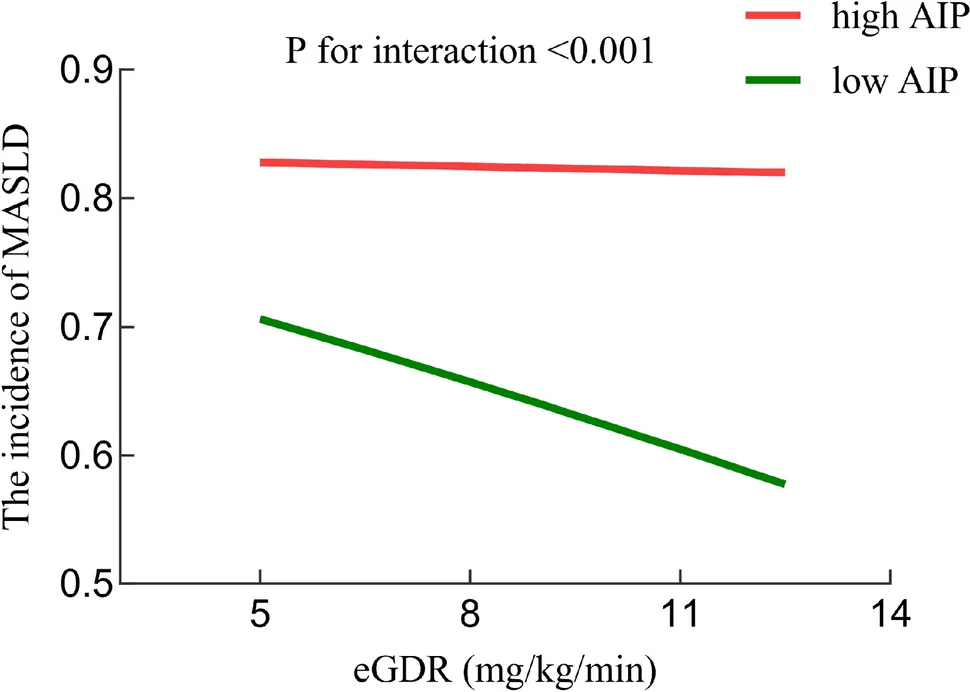
Interaction between eGDR and MASLD Risk at Different AIP Levels. Adjusted for the integrated Model 3 (age, sex, BMI, FBG, LDL-C, hepatic and renal functions, smoking and drinking status). *AIP* atherogenic index of plasma, *eGDR* estimated glucose disposal rate, *MASLD* metabolic dysfunction-associated steatotic liver disease

We identified a significant multiplicative interaction between eGDR and AIP (P for interaction < 0.001; Table 3; interaction term OR = 1.17, 95% CI 1.10–1.24). This interaction represented a clinically important effect modification. The significant interaction was further elucidated by visualization (Fig. 2). Consistent with the primary negative association, a higher eGDR was linked to a lower risk of MASLD. However, this inverse association appeared substantially modified by AIP levels: it was most pronounced among individuals with a low AIP and became progressively less evident as AIP increased. Critically, the predicted risk curves for low and high AIP converged at lower eGDR values. This pattern suggests that, in this cross-sectional analysis, the difference in MASLD risk associated with varying AIP levels narrows at lower eGDR values, consistent with a convergence of risk associations rather than an additive relationship. This convergent pattern was preserved in sensitivity analyses with additional adjustment for the constituent metabolic variables (Supplemental Fig. 3).

### Exploratory mediation analysis

**Fig. 3:**
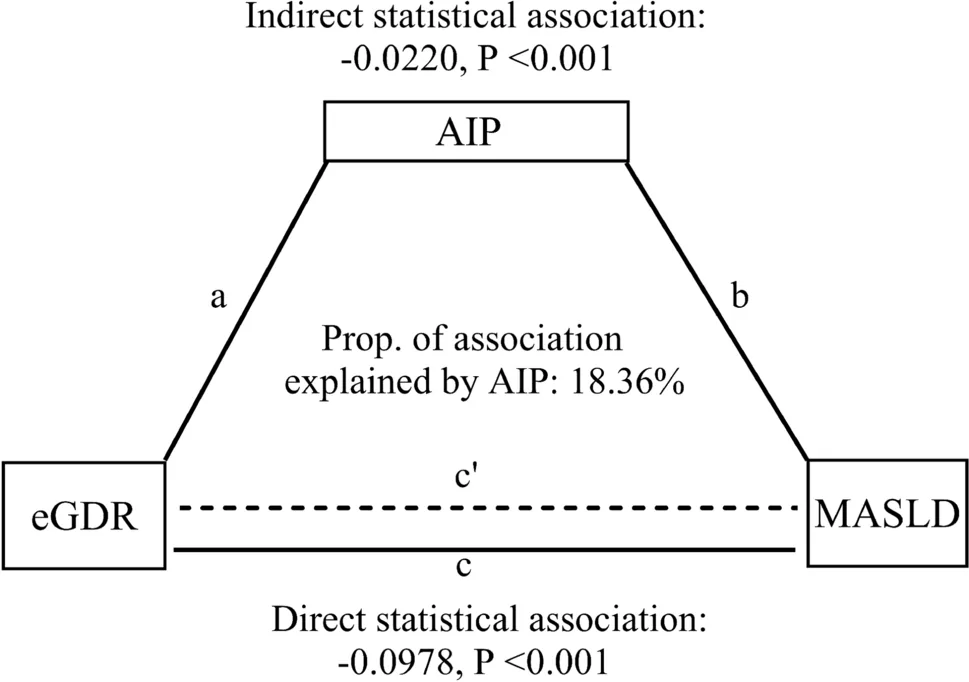
Path diagram of the statistical associations between eGDR, AIP, and MASLD. Non-directional lines represent statistical associations assessed in a cross-sectional framework and do not imply temporal causality. In this diagram, the values adjacent to the lines represent standardized regression coefficients from the fitted models: **a** the statistical association between eGDR and AIP; **b** the statistical association between AIP and MASLD adjusting for eGDR; **c** the total statistical association between eGDR and MASLD before accounting for AIP; and (c') the remaining statistical association between eGDR and MASLD after accounting for AIP. All analyses were adjusted for the integrated Model 3 (age, sex, BMI, FBG, LDL-C, hepatic and renal functions, smoking and drinking status). *AIP* atherogenic index of plasma, *eGDR* estimated glucose disposal rate, *MASLD* metabolic dysfunction-associated steatotic liver disease

To explore whether the atherogenic lipid profile statistically aligns with part of the relationship between insulin resistance and MASLD in this dataset, we performed an exploratory mediation analysis with eGDR as the exposure, AIP as the intermediary, and MASLD as the outcome. It is critical to note that this analysis does not establish temporal or causal mediation due to the cross-sectional design; rather, it quantifies the degree to which the association between eGDR and MASLD overlaps with AIP in this dataset. The analysis revealed a significant indirect statistical pathway (Fig. 3). Lower eGDR was associated with a significant increase in AIP (a-path), which in turn was strongly associated with a higher risk of MASLD (b-path). The bootstrap-estimated indirect association was −0.0220 (p < 0.001), while the direct association of eGDR on MASLD was −0.0978 (p < 0.001). In this cross-sectional framework, approximately 18.4% of the statistical association between eGDR and MASLD was accounted for by AIP. It is critical to emphasize that this analysis assesses covariation patterns and does not establish causal mediation or temporal sequence.

### Risk stratification based on combined metabolic phenotypes

Based on the inflection points from the RCS curves in the full cohort (eGDR = 8.0 mg/kg/min, AIP = –0.15), participants were classified into four metabolic phenotypes (Table 4). The crude prevalence of MASLD increased stepwise across the four phenotypes: 13.30% in Phenotype I, 37.26% in Phenotype II, 53.32% in Phenotype III, and 73.87% in Phenotype IV. Compared to the reference phenotype (Phenotype I), the adjusted OR for MASLD was 1.31 (95% CI 1.12–1.53) for Phenotype II, 2.90 (95% CI 2.63–3.18) for Phenotype III, and 3.42 (95% CI 3.07–3.81) for Phenotype IV. The risk gradient starkly illustrated the clinical implication of the interaction. Most notably, the incremental risk associated with adding high AIP to a state of low eGDR (from Phenotype III to IV, ORΔ ≈ 0.52) was far smaller than the risk difference incurred by moving from high to low eGDR within a favorable lipid milieu (from Phenotype I to III, ORΔ = 1.90). This quantitatively mirrors the graphical convergence observed in Fig. 2.

**Table 4:** Association between the metabolic phenotypes and MASLD risk

Metabolic phenotype	No	Crude prevalence of MASLD (%)	Model 1 OR (95% CI)	Model 2 OR (95% CI)	Model 3 OR (95% CI)
I	6609	13.30	Ref	Ref	Ref
II	1503	37.26	3.87 (3.41–4.39)	3.47 (3.04–3.96)	1.31 (1.12–1.53)
III	14,029	53.32	7.45 (6.88–8.05)	6.33 (5.84–6.86)	2.90 (2.63–3.18)
IV	8002	73.87	18.43 (16.90–20.10)	15.56 (14.21–17.04)	3.42 (3.07–3.81)

Model 1: unadjusted. Model 2: adjusted for age and sex. Model 3 (the integrated model 3): adjusted for age, sex, BMI, FBG, LDL-C, hepatic and renal functions, smoking and drinking status

Phenotype thresholds (eGDR = 8.0 mg/kg/min, AIP = –0.15) were derived from RCS inflection points in the full cohort. To address potential overfitting (“double-dipping”), we performed an internal split-sample validation: thresholds were re-estimated in a random training set (70% of the cohort) and then applied to an independent validation set (30% of the cohort). The validation set confirmed the robustness of the risk gradient (Supplemental Table 4). External validation in independent cohorts is required before clinical application of these specific numeric cut-offs

Crude prevalence of MASLD (%) is unadjusted and represents absolute risk

*CI* confidence interval, *MASLD* metabolic dysfunction-associated steatotic liver disease, *OR* odds ratio, Phenotype I (high-eGDR/low-AIP); Phenotype II (high-eGDR/high-AIP); Phenotype III (low-eGDR/low-AIP); Phenotype IV (low-eGDR/high-AIP)

To address the potential for overfitting and type I error inflation (“double-dipping”), we performed an internal split-sample validation. The RCS-derived thresholds were re-estimated in a randomly selected training set (70% of the cohort) and then applied to an independent validation set (30% of the cohort). As shown in Supplemental Table 4, the validation set produced a highly consistent stepwise increase in MASLD risk: the adjusted ORs for Phenotypes II, III, and IV were 1.28 (95% CI 1.14–1.47), 3.00 (95% CI 2.66–3.42), and 3.78 (95% CI 3.29–4.27), respectively. The risk ordering (II < III < IV) and overlapping confidence intervals between the training and validation sets confirm that the findings are robust and not an artifact of threshold optimization.

Furthermore, sensitivity analyses that additionally adjusted for the constituent variables of eGDR and AIP did not materially alter the associations (Supplemental Tables 2a, b), indicating that the observed effects are not merely driven by these individual components. Critically, the median-split analysis (Supplemental Table 3) yielded a fully concordant, stepwise increase in MASLD risk with an identical ordinal ranking (median-based OR for the combined adverse phenotype = 3.98, 95% CI 3.62–4.37). This consistency across different cut-point definitions supports the robustness of the risk stratification framework, although the specific numeric thresholds from the RCS analysis remain exploratory and require external validation.

### Sensitivity analyses

Sensitivity analyses affirmed the robustness of the primary findings. First, to address potential overadjustment and isolate the integrative value of the composite indices, we performed analyses that adjusted for their constituent metabolic variables (eGDR: HbA1c, hypertension, BMI; AIP: TG, HDL-C). Despite this stringent correction, which absorbed substantial shared variance, the strong, graded associations of both eGDR and AIP with MASLD risk remained highly significant across quartiles and as continuous variables (all p < 0.001; Supplemental Tables 2a, b). Critically, the central graphical findings—the nonlinear dose–response curves (Supplemental Fig. 2), the convergent interaction pattern (Supplemental Fig. 3), and the significant statistical alignment pathway (Supplemental Fig. 4)—were all rigorously reproduced in these adjusted models, confirming their stability beyond individual components.

Second, to evaluate the dependence of risk stratification on specific thresholds, we redefined metabolic phenotypes using median splits of eGDR and AIP instead of RCS-derived inflection points. The results were qualitatively unchanged, demonstrating a fully concordant, stepwise increase in MASLD risk with an identical ordinal ranking (Supplemental Table 3). The high-risk profile for the combined adverse phenotype was consistently replicated (median-based OR = 3.98, 95% CI 3.62–4.37 vs. RCS-based OR = 3.42, 95% CI 3.07–3.81; both p < 0.001). Collectively, these analyses confirm that the core associations, interaction, and risk stratification are not artifacts of model specification, overadjustment, or cut-point selection. This median-split analysis provided a conservative check of the risk gradient independent of the RCS-derived thresholds.

## Discussion

Beyond confirming eGDR and AIP as independent risk markers for MASLD, this study reveals a non-additive interaction characterized by “risk convergence”. This statistical pattern generates the hypothesis that the contribution of atherogenic dyslipidemia to liver fat accumulation may appear diminished when insulin resistance becomes severe, thereby challenging a model of simple metabolic risk summation. While the cross-sectional design precludes causal inference, this interaction directly informs a stratification tool to identify a high-risk phenotype (Phenotype III) that warrants prospective validation and may be potentially overlooked by conventional lipid screening.

Our findings confirm the strong, independent, and non-linear associations of lower eGDR and higher AIP with MASLD risk, aligning with prior literature [8, 15, 16]. For instance, a recent cross-sectional analysis demonstrated significant inverse associations of eGDR with both MASLD (OR = 0.53) and liver fibrosis (OR = 0.40) [15]. Concurrently, higher AIP has been robustly established as a predictor for both the incident risk of MASLD [8] and, as evidenced by a U.S. cohort study, its prognosis [16]. However, previous studies have largely examined these core metabolic pathways—insulin sensitivity and atherogenic dyslipidemia—in isolation. A key advance of our study is the quantitative demonstration, in a large clinical cohort, of a significant statistical interaction between eGDR and AIP regarding MASLD risk. To our knowledge, this is the first study to explicitly model and report this interaction, revealing a “risk convergence” pattern that challenges a purely additive model of metabolic risk.

The observed risk convergence—where the marginal risk contribution of atherogenic dyslipidemia (high AIP) appears diminished under conditions of severe insulin resistance (low eGDR)—requires cautious interpretation given the cross-sectional nature of our data. While the statistical interaction is robust, it generates hypotheses regarding underlying physiology rather than providing direct evidence. We hypothesize that this pattern may reflect a shift in hepatic lipid source dominance. In severe insulin resistance, hepatic lipid accumulation may be predominantly driven by hyperinsulinemia-stimulated de novo lipogenesis (DNL) and increased adipose tissue lipolysis [17, 18], which could proportionally reduce the relative contribution from circulating atherogenic particles reflected by AIP. Alternatively, severe insulin resistance and chronic inflammation are often concomitant [19, 20], the latter being a key driver of MASLD that promotes progression to metabolic dysfunction-associated steatohepatitis (MASH) and modulates lipid metabolism [21, 22]. Pro-inflammatory cytokines can disrupt lipid handling and impair insulin signaling [23, 24], suggesting that low eGDR and high AIP may both serve as markers of a dominant inflammatory drive, potentially obscuring their independent statistical effects in this cross-sectional view. These proposed mechanisms are speculative and require validation in longitudinal studies with detailed phenotyping. Importantly, our mediation analysis, while statistically significant, cannot distinguish causality from confounding by unmeasured factors such as visceral adiposity or systemic inflammation. Third, individual genetic predispositions may set a threshold for hepatocellular lipid handling, beyond which inputs from various sources lead to similar injury outputs [25]. Notably, this interactive risk pattern may extend beyond hepatic steatosis. Since chronic inflammation heightens atherosclerosis risk [26], the confluence of low eGDR and high AIP could further amplify cardiovascular mortality risk in MASLD, warranting future investigation. These proposed mechanisms are not mutually exclusive and may operate concurrently to shape the observed risk convergence landscape. Their relative contributions and interactions warrant further investigation through longitudinal studies integrating multi-omics approaches and detailed metabolic phenotyping.

This dynamic interaction provides a foundation for exploring refined clinical risk stratification. The metabolic phenotype model, based on the interaction-derived thresholds (eGDR = 8.0 mg/kg/min, AIP = −0.15), offers a practical tool for risk stratification. Phenotype III (low-eGDR/low-AIP) is of particular clinical relevance. Despite the absence of significant atherogenic dyslipidemia, these individuals harbor severe insulin resistance and carry a MASLD risk (OR = 2.90) approaching that of the combined dysfunction group (phenotype IV, OR = 3.42). In absolute terms, the crude prevalence of MASLD in Phenotype III was 53.32%—more than four times higher than the 13.30% observed in the reference phenotype (Phenotype I)—highlighting that even without lipid abnormalities, severe insulin resistance confers a substantial absolute risk. Thus, the adjusted ORs should be interpreted alongside these absolute risks to fully appreciate the clinical magnitude. If validated in prospective cohorts, this phenotype, which may overlap with conditions like “metabolically unhealthy normal weight” [27, 28] could represent an “insulin-sensitivity-first” high-risk profile that might be missed in routine screening focused primarily on lipid profiles [29]. Therefore, our data suggest a potential role for incorporating eGDR into routine health checks —easily calculated from existing WC, HbA1c, and hypertension data— as a hypothesis-generating approach to facilitate the early detection of such individuals with concealed metabolic risk. Although the specific thresholds proposed here (eGDR = 8.0 mg/kg/min, AIP = −0.15) require external validation, the consistent risk ordering observed with median-based cut-points partly supports the generalizability of the four-phenotype framework. This pragmatic stratification could be readily implemented in health check-up settings or primary care, using existing laboratory data to flag individuals requiring further metabolic evaluation or intensified management, pending prospective validation.

Notably, the four-quadrant phenotyping itself provides an intuitive visualization of the core interaction. The gradient of risk estimates increase provides quantitative illustration for convergence: the difference in MASLD odds between Phenotype IV and Phenotype III (ΔOR ≈ 0.52) was substantially smaller than the difference between Phenotype III and Phenotype I (ΔOR = 1.90). While causal inference is precluded by the cross-sectional design, this pattern generates the hypothesis that, for individuals with low eGDR (Phenotypes III and IV), the MASLD risk observed in this cohort was elevated irrespective of AIP level. If confirmed in prospective studies, this could suggest that initial therapeutic efforts targeting insulin sensitivity might warrant prioritized investigation in such individuals. At present, these findings only support the need for further research into phenotype-guided management strategies, consistent with the comprehensive framework recommended by guidelines [12, 30].

Furthermore, our exploratory mediation analysis suggests a statistical overlap whereby AIP accounts for approximately 18.4% of the observed association between eGDR and MASLD. This provides partial statistical support for the pathophysiological sequence wherein insulin resistance exacerbates atherogenic dyslipidemia, thereby contributing to hepatic steatosis [31]. However, caution is warranted in interpretation. Given the cross-sectional design, this analysis reveals a plausible pattern of statistical covariation but cannot distinguish between alternative causal models, reverse causation, or confounding by unmeasured factors. The substantial remaining direct statistical association (81.6%) underscores that eGDR likely captures additional pathways beyond those reflected by AIP.

The clinical translation of these findings provides a rationale for future research into phenotype-guided risk assessment, rather than an immediate mandate for clinical practice. Integrating the readily available eGDR and AIP metrics could inform initial stratification in primary care or health check-up settings in future prospective studies. The key insight from our study is the potential reprioritization of clinical focus: For individuals with low eGDR (Phenotypes III and IV), clinical decision-making might be strongly centered on assessing and improving insulin sensitivity, irrespective of AIP level. Our phenotypic framework provides a simple tool to identify candidates for further investigation of this “insulin-sensitivity-first” assessment strategy. A potential clinical implementation algorithm for future validation could involve: (1) automated calculation of eGDR and AIP from electronic health records during routine check-ups; (2) classification of individuals into the four metabolic phenotypes; (3) for those in Phenotypes III and IV (low eGDR), triggering prioritized interventions focused on improving insulin sensitivity (e.g., structured lifestyle programs, consideration of insulin-sensitizing pharmacotherapy), while for Phenotype II, targeted management of dyslipidemia remains key alongside general metabolic health promotion. For Phenotype IV (combined risk), early combined intervention remains warranted. The prospective utility and impact on outcomes of this stratification require validation in longitudinal and intervention studies.

We acknowledge that the confidence interval for the main effect of eGDR widened in the fully adjusted model, reflecting the stringent statistical control for correlated metabolic variables. Critically, the multiplicative interaction term (eGDR × AIP) remained statistically significant, supporting the robustness of the non-additive relationship. Furthermore, while the visual convergence of risk curves in Fig. 2 provides a descriptive illustration of the interaction pattern, the formal inference of effect modification is based on the logistic regression product term, thereby mitigating concerns regarding ecological fallacy. Nonetheless, the reduced precision of the main effect estimate underscores that the exact magnitude of the association observed in this single-center cohort should be interpreted with caution, and its quantitative generalizability to populations with differing metabolic characteristics requires confirmation in external studies.

Limitations of our study must be acknowledged. First, the cross-sectional design fundamentally precludes causal inference, and the observed interaction may be susceptible to reverse causation. Second, both core indices are surrogate measures from single-time-point assessments, and unaccounted medication effects (e.g., lipid-lowering drugs) may influence the results. Third, the diagnosis of MASLD via ultrasonography lacks sensitivity for mild steatosis [32] and cannot distinguish metabolic-related steatosis from other etiologies or assess histological severity. The single-center, health-examination-based cohort may entail selection bias, limiting the generalizability of the absolute risk thresholds. Fourth, as with any analysis of real-world health screening data, the requirement for complete case data may introduce selection bias. In this cohort, the primary reason for exclusion was non-selection of optional abdominal ultrasonography rather than clinical contraindication. Although comparisons of baseline characteristics between the final analytic cohort and excluded participants due to incomplete data showed largely balanced distributions of key metabolic covariates (SMD < 0.1 for all variables), we cannot entirely exclude the possibility of residual selection bias. Consequently, the reported associations should be interpreted with appropriate caution, and the generalizability of the absolute risk thresholds to populations with more advanced metabolic disease may be limited. Fifth, we explicitly acknowledge the potential for incorporation bias. Given that the diagnostic criteria for MASLD share conceptual domains with components of the exposure indices, the observed associations may be partially inflated. While this limitation cannot be fully eliminated, we adopted a two-pronged approach to mitigate its impact. First, our primary adjusted models excluded the direct constituent variables of eGDR and AIP to minimize mathematical coupling. Second, sensitivity analyses that re-introduced these constituent variables as covariates showed that the associations, while modestly attenuated, remained statistically significant. This supports the view that eGDR and AIP function as integrative biomarkers of the severity of the underlying metabolic dysfunction, rather than as mere proxies for the diagnostic criteria. Nonetheless, the reported effect sizes should be interpreted with caution and represent the upper bound of the association. In addition, we recognize that deriving phenotype thresholds in the full cohort from RCS inflection points carries a risk of overfitting and may inflate type I error (“double-dipping”). To mitigate this for the four-phenotype classification, we performed an internal split-sample validation (training set 70%, validation set 30%), which confirmed the robustness of the risk gradient. However, this internal validation does not substitute for external validation in independent cohorts. The specific numeric cut-offs should therefore be viewed as hypothesis-generating until validated in other populations. Most critically, we lacked data on key potential confounders including visceral adiposity (a core driver of both insulin resistance and inflammation), detailed dietary patterns, objectively measured physical activity, and sleep quality. The observed statistical interaction could therefore still be confounded by these powerful upstream shared determinants. Finally, the proposed mechanistic interpretations—including the hypothesized saturation of hepatic lipid uptake in severe insulin resistance—remain post-hoc hypotheses derived from statistical patterns observed in cross-sectional data. Importantly, the mediation analysis, while insightful in describing the data structure, cannot distinguish the proposed causal pathway from alternative models involving reverse causation or confounding by unmeasured factors like visceral fat.

Nevertheless, the consistency of the core findings across multiple sensitivity analyses underscores the robustness of the observed statistical interaction and provides a substantive foundation for further causal investigation.

## Conclusion

This study demonstrates that lower eGDR and higher AIP are independently and interactively associated with increased MASLD risk, following a convergent pattern: the incremental risk associated with atherogenic dyslipidemia appears diminished in the setting of severe insulin resistance. Their combination identifies a distinct high-risk phenotype. If validated prospectively, integrating eGDR and AIP, two simple routine metrics, may inform early MASLD risk stratification by accounting for their dynamic interaction. Prospective studies are warranted to validate the predictive utility of this phenotypic framework, elucidate its underlying mechanisms, and evaluate its impact on clinical decision-making and patient outcomes.

### Impact and implications

This cross-sectional study provides evidence of a non-additive, convergent interaction between insulin resistance (estimated glucose disposal rate, eGDR) and atherogenic dyslipidemia (atherogenic index of plasma, AIP) in their association with metabolic dysfunction-associated steatotic liver disease (MASLD) risk. The findings generate the hypothesis that severe insulin resistance confers high MASLD risk even in the absence of overt dyslipidemia—a phenotype potentially missed by lipid-focused screening alone. The proposed four-phenotype framework, derived from routine clinical parameters, offers a practical tool for risk stratification in primary care or health check-up settings, potentially enabling more targeted, phenotype-guided interventions. However, given the cross-sectional design and single-center cohort, these findings should be interpreted as hypothesis-generating. Prospective validation and mechanistic studies are needed before clinical implementation, and the proposed thresholds should be interpreted cautiously in other populations.

## Supplementary Information

Below is the link to the electronic supplementary material.

Supplementary file1 (DOCX 5821 KB)

## Data Availability

The datasets used and/or analyzed during the current study are available from the corresponding author on reasonable request.

## Abbreviations

AIP: Atherogenic index of plasma
ALT: Alanine aminotransferase
AST: Aspartate transaminase
BMI: Body mass index
BUN: Blood urea nitrogen
CI: Confidence interval
Cr: Creatinine
DBP: Diastolic blood pressure
eGDR: Estimated glucose disposal rate
FBG: Fasting blood glucose
GGT: γ‐Glutamyltransferase
HbA1c: Glycosylated hemoglobin
HDL-C: High-density lipoprotein cholesterol
IQR: Interquartile range
LDL-C: Low-density lipoprotein cholesterol
MASLD: Metabolic dysfunction-associated steatotic liver disease
OR: Odds ratio
RCS: Restricted cubic splines
SBP: Systolic blood pressure
TC: Total cholesterol
TG: Triglyceride
UA: Uric acid
WC: Waist circumference
