# Supplementary material for: Converging pathways: the interaction between estimated glucose disposal rate and atherogenic index of plasma in MASLD risk stratification
Source: Hepatol Int. 2026 May 25;20(5):1340–52. doi: 10.1007/s12072-026-11098-0 (PMC13676677; doi:10.1007/s12072-026-11098-0)
Supplement: Supplementary file 1 [file 12072_2026_11098_MOESM1_ESM.docx]

**Supplemental Table 1. Comparison of baseline characteristics between the final analytic cohort and participants excluded due to incomplete abdominal ultrasonography data.**

| **Characteristics** | **Overall**  **(n=30,143)** | **Excluded Cohort**  **(n=9,291)** | **SMD** |
| --- | --- | --- | --- |
| **General information** | | | |
| Male *^a^* | 21,105 (70.01) | 5,928 (63.80) | 0.132 |
| Age, year *^b^* | 49 (38, 57) | 49 (39, 57) | 0.004 |
| BMI, kg/m^2^ *^c^* | 25.25 ± 3.50 | 25.19 ± 3.52 | 0.017 |
| WC, cm *^c^* | 85.90 ± 10.76 | 85.11 ± 10.86 | 0.073 |
| SBP, mmHg *^c^* | 126.31 ± 17.62 | 126.85 ± 17.25 | 0.031 |
| DBP, mmHg *^c^* | 78.81 ± 12.56 | 78.15 ± 11.78 | 0.054 |
| **Glucolipid metabolism** | | | |
| FBG, mmol/L *^b^* | 5.27 (4.94, 5.73) | 5.21 (4.88, 5.67) | 0.023 |
| HbA1c, % *^b^* | 5.6 (5.3, 5.9) | 5.6 (5.4, 5.9) | 0.020 |
| TC, mmol/L *^c^* | 4.87 ± 0.99 | 4.95 ± 0.99 | 0.081 |
| TG, mmol/L *^b^* | 1.41 (0.97, 2.12) | 1.42 (0.98, 2.13) | 0.009 |
| HDL-C, mmol/L *^b^* | 1.28 (1.10, 1.50) | 1.30 (1.12, 1.52) | 0.035 |
| LDL-C, mmol/L *^c^* | 3.02 ± 0.80 | 2.98 ± 0.70 | 0.053 |
| **Hepatic and Renal function** | | | |
| ALT, U/L *^b^* | 21 (15, 32) | 21 (15, 31) | 0.022 |
| AST, U/L *^b^* | 21 (17, 25) | 20 (17, 25) | 0.014 |
| GGT, U/L *^b^* | 26 (18, 42) | 26 (17, 41) | 0.012 |
| BUN, mmol/L *^b^* | 5.02 (4.25, 5.93) | 4.84 (4.10, 5.72) | 0.045 |
| Cr, μmol/L *^b^* | 69 (59, 78) | 68 (58, 76.25) | 0.017 |
| UA, μmol/L *^b^* | 332 (272, 394) | 329 (271, 390) | 0.012 |
| **Index** | | | |
| eGDR, mg/kg/min *^b^* | 9.86 (7.11, 10.91) | 10.01 (7.28, 11.04) | 0.042 |
| AIP *^b^* | 0.04 (-0.17, 0.26) | 0.04 (-0.16, 0.25) | 0.002 |
| **Behavioral risk factors** | | | |
| Current smoker *^a^* | 4,685 (15.54) | 1,393 (14.99) | 0.015 |
| Former smoker *^a^* | 8,584 (28.48) | 2,602 (28.00) | 0.011 |
| Never smoker *^a^* | 16,874 (55.97) | 5,229 (56.28) | 0.006 |
| Mild drinker *^a^* | 19,266 (63.92) | 5,901 (63.51) | 0.008 |
| Never drinker *^a^* | 10,877 (36.08) | 3,342 (35.97) | 0.002 |

^a^Data are presented as n (%). ^b^Data are presented as median (interquartile range, IQR). ^c^Data are presented as mean (SD).

An SMD < 0.1 was considered indicative of negligible imbalance between groups. The excluded cohort comprises participants excluded solely due to incomplete abdominal ultrasonography data for MASLD diagnosis (n=9,291); individuals excluded for specific medical contraindications (n=551) and incomplete questionnaire (n=288) were not included in this comparison.

Abbreviations: AIP, atherogenic index of plasma; ALT, alanine aminotransferase; AST, aspartate transaminase; BMI, body mass index; BUN, blood urea nitrogen; Cr, creatinine; DBP, diastolic blood pressure; eGDR, estimated glucose disposal rate; FBG, fasting blood glucose; GGT, γ‐glutamyltransferase; HbA1c, glycosylated hemoglobin; HDL-C, high-density lipoprotein cholesterol; LDL-C, low-density lipoprotein cholesterol; SMD, standardized mean differences; MASLD, metabolic dysfunction-associated steatotic liver disease; SBP, systolic blood pressure; TC, total cholesterol; TG, triglyceride; UA, uric acid; WC, waist circumference.

#### ****Supplemental Table 2a. Sensitivity analysis of the associations between eGDR/AIP quartiles and MASLD risk after additional adjustment for constituent variables****

| **Exposure / Phenotype** | **Primary analysis (Model 3)** | | **Sensitivity analysis (Model 3 + Components)** | |
| --- | --- | --- | --- | --- |
|  | **OR (95% CI)** | **P value** | **OR (95% CI)** | **P value** |
| **eGDR quartiles** | | | | |
| Q4 | Ref. | / | Ref. | / |
| Q3 | 2.25 (2.02-2.51) | <0.001 | 1.76 (1.55-1.99) | <0.001 |
| Q2 | 2.68 (2.38-3.02) | <0.001 | 1.86 (1.55-2.23) | <0.001 |
| Q1 | 2.91 (2.56-3.30) | <0.001 | 1.93 (1.44-2.58) | <0.001 |
| **AIP quartiles** | | | | |
| Q1 | Ref. | / | Ref. | / |
| Q2 | 1.83 (1.67-2.01) | <0.001 | 1.76 (1.59-1.94) | <0.001 |
| Q3 | 3.10 (2.82-3.41) | <0.001 | 2.80 (2.51-3.13) | <0.001 |
| Q4 | 6.03 (5.45-6.68) | <0.001 | 4.55 (3.89-5.33) | <0.001 |

Primary analysis: Model 3 was fully adjusted for independent association. For eGDR models, WC, hypertension, HbA1c, and TC were excluded; and for AIP models, TG, HDL-C, and BMI were excluded.

Sensitivity analysis: Model 3 + the constituent variables of the respective index (WC, hypertension, HbA1c for eGDR; TG, HDL-C for AIP).

Abbreviations: AIP, atherogenic index of plasma; CI, confidence interval; eGDR, estimated glucose disposal rate; MASLD, metabolic dysfunction-associated steatotic liver disease; OR, odds ratio.

#### ****Supplemental Table 2b. Sensitivity analysis of the associations of continuous eGDR/AIP, their interaction, and derived metabolic phenotypes with MASLD risk****

| **Exposure / Phenotype** | **Primary analysis (Model 3)** | | **Sensitivity analysis (Model 3 + Components)** | |
| --- | --- | --- | --- | --- |
|  | **OR (95% CI)** | **P value** | **OR (95% CI)** | **P value** |
| **eGDR (per 1-IQR decrease)** | 1.78 (1.23, 2.58) | 0.004 | 2.52 (1.53, 4.16) | <0.001 |
| **AIP (per 1-IQR increase)** | 9.50 (8.32, 10.84) | <0.001 | 4.03 (3.61, 4.50) | <0.001 |
| **eGDR×AIP interaction** | 1.17 (1.10, 1.24) | <0.001 | 1.12 (1.06, 1.19) | <0.001 |
| **Metabolic phenotype** | | | | |
| Ι | Ref. | / | Ref. | / |
| Π | 1.31 (1.12-1.53) | <0.001 | 1.20 (1.03-1.41) | <0.001 |
| Ⅲ | 2.90 (2.63-3.18) | <0.001 | 1.88 (1.69-2.09) | <0.001 |
| Ⅳ | 3.42 (3.07-3.81) | **<0.001** | 1.98 (1.75-2.24) | <0.001 |

Primary analysis was adjusted for the integrated Model 3 (age, sex, BMI, FBG, LDL-C, hepatic and renal functions, smoking and drinking status).

Sensitivity analysis was adjusted for the integrated Model 3 + constituent variables of both eGDR (WC, hypertension, HbA1c) and AIP (TG, HDL-C).

The interaction term is the product of mean-centered eGDR and AIP.

Phenotypes are defined using RCS-derived inflection points (eGDR=8.0 mg/kg/min, AIP=-0.15).

#### ****Supplemental Table 3. Comparison of metabolic phenotype definitions: RCS-derived inflection points versus median splits****

| **Metabolic**  **phenotype** | **Using RCS-derived inflection points**  **(eGDR=8.0, AIP=-0.15)** | | **Using median splits**  **(eGDR=9.86, AIP=0.04)** | |
| --- | --- | --- | --- | --- |
|  | **OR (95% CI)** | **P value** | **OR (95% CI)** | **P value** |
| Ι | Ref. | / | Ref. | / |
| Π | 1.31 (1.12-1.53) | <0.001 | 1.63 (1.47-1.79) | <0.001 |
| Ⅲ | 2.90 (2.63-3.18) | <0.001 | 3.14 (2.87-3.43) | <0.001 |
| Ⅳ | 3.42 (3.07-3.81) | **<0.001** | 3.98 (3.62-4.37) | <0.001 |

All analyses were adjusted for the integrated Model 3 (age, sex, BMI, FBG, LDL-C, hepatic and renal functions, smoking and drinking status).

Abbreviations: CI, confidence interval; MASLD, metabolic dysfunction-associated steatotic liver disease; OR, odds ratio; RCS, restricted cubic spline.

| **Metabolic phenotype** | **No.** | **Crude prevalence of MASLD (%)** | **Model 1**  **OR (95% CI)** | **Model 2**  **OR (95% CI)** | **Model 3**  **OR (95% CI)** |
| --- | --- | --- | --- | --- | --- |
| **Training set (70% of cohort)** | | | | | |
| Ι | 5912 | 11.89 | Ref. | Ref. | Ref. |
| Π | 2408 | 42.19 | 4.53 (4.13-5.09) | 4.86 (4.23-5.11) | 1.37 (1.26-1.58) |
| Ⅲ | 4714 | 48.83 | 7.07 (6.42-7.79) | 5.99 (5.04-6.57) | 2.88 (2.65-3.17) |
| Ⅳ | 8065 | 79.19 | 22.20 (19.64-24.59) | 17.92 (16.44-19.88) | 3.50 (3.12-3.91) |
| **validation set (30% of cohort)** | | | | | |
| Ι | 2526 | 11.20 | Ref. | Ref. | Ref. |
| Π | 1058 | 44.71 | 4.22 (3.84-4.69) | 3.46 (2.93-3.68) | 1.28 (1.14-1.47) |
| Ⅲ | 2022 | 47.73 | 8.35 (7.41-8.96) | 7.05 (6.46-7.60) | 3.00 (2.66-3.42) |
| Ⅳ | 3438 | 78.56 | 23.44 (21.06-26.30) | 18.00 (16.58-20.10) | 3.78 (3.29-4.27) |

**Supplemental Table 4. Internal split‑sample validation of the four‑phenotype classification for MASLD risk**

Model 1: unadjusted. Model 2: adjusted for age and sex. Model 3 (the integrated Model 3): adjusted for age, sex, BMI, FBG, LDL-C, hepatic and renal functions, smoking and drinking status.
RCS-derived thresholds (eGDR = 8.0 mg/kg/min, AIP = –0.15) were re‑estimated in the training set (70% of the cohort) and then applied to both the training set and the validation set (30% of the cohort).
Crude prevalence is unadjusted and represents absolute risk.

This internal validation helps to address the concern of double‑dipping; however, we acknowledge that external validation in independent cohorts would be valuable to further strengthen the generalizability of these findings.

Abbreviations: CI, confidence interval; MASLD, metabolic dysfunction-associated steatotic liver disease; OR, odds ratio; Phenotype I (high-eGDR/low-AIP); Phenotype II (high-eGDR/high-AIP); Phenotype III (low-eGDR/low-AIP); Phenotype IV (low-eGDR/high-AIP).

**Supplemental Figure 1** Study flow diagram

**(b)**

**(a)**

**(d)**

**(c)**

**(d)**

**(c)**

**Supplemental Figure 2. Sensitivity analysis of the dose-response relationships of eGDR and AIP with MASLD risk using restricted cubic splines.**

**(a)** eGDR and MASLD, Primary Analysis.
**(b)** eGDR and MASLD, Sensitivity Analysis.
**(c)** AIP and MASLD, Primary Analysis.
**(d)** AIP and MASLD, Sensitivity Analysis.

Primary analysis was adjusted for the integrated Model 3 (age, sex, BMI, FBG, LDL-C, hepatic and renal functions, smoking and drinking status).

Sensitivity analysis was adjusted for the integrated Model 3 + constituent variables of both eGDR (WC, hypertension, HbA1c) and AIP (TG, HDL-C).

**The non-linear associations and risk inflection points remained evident after stringent adjustment for the constituent variables of eGDR and AIP, confirming the robustness of the observed thresholds.**

**Supplemental Figure 3. Sensitivity analysis of the interaction between eGDR and MASLD risk stratified by AIP levels.**

(Left) Adjusted using the Primary Analysis.
(Right) Adjusted using the Sensitivity Analysis.

Primary analysis was adjusted for the integrated Model 3 (age, sex, BMI, FBG, LDL-C, hepatic and renal functions, smoking and drinking status).

Sensitivity analysis was adjusted for the integrated Model 3 + constituent variables of both eGDR (WC, hypertension, HbA1c) and AIP (TG, HDL-C).
**The convergent pattern—where the predicted risk curves for low and high AIP strata approach each other at low eGDR values—is consistent across both adjustment strategies, supporting the robustness of the observed interaction.**

**Supplemental Figure 4. Sensitivity analysis of statistical associations between eGDR, AIP, and MASLD.**

(Left) Diagram of statistical associations based on the Primary Analysis.
(Right) Diagram of statistical associations based on the Sensitivity Analysis.

Primary analysis was adjusted for the integrated Model 3 (age, sex, BMI, FBG, LDL-C, hepatic and renal functions, smoking and drinking status).

Sensitivity analysis was adjusted for the integrated Model 3 + constituent variables of both eGDR (WC, hypertension, HbA1c) and AIP (TG, HDL-C). **All coefficients for the statistical pathways remained statistically significant (P < 0.05) in both models, demonstrating the consistency of the observed pattern of associations. Non‑directional lines represent statistical associations assessed in a cross‑sectional framework and do not imply temporal causality.**
